# mRNA Sonotransfection of Tumors with Polymeric Microbubbles: Co‐Formulation versus Co‐Administration

**DOI:** 10.1002/advs.202306139

**Published:** 2024-02-11

**Authors:** Junlin Chen, Bi Wang, Yuchen Wang, Harald Radermacher, Jinwei Qi, Jeffrey Momoh, Twan Lammers, Yang Shi, Anne Rix, Fabian Kiessling

**Affiliations:** ^1^ Institute for Experimental Molecular Imaging Helmholtz Institute for Biomedical Engineering RWTH Aachen University 52074 Aachen Germany

**Keywords:** co‐administration, co‐formulation, focused ultrasound, lipoplexes, mRNA delivery, PBCA microbubbles

## Abstract

Despite its high potential, non‐viral gene therapy of cancer remains challenging due to inefficient nucleic acid delivery. Ultrasound (US) with microbubbles (MB) can open biological barriers and thus improve DNA and mRNA passage. Polymeric MB are an interesting alternative to clinically used lipid‐coated MB because of their high stability, narrow size distribution, and easy functionalization. However, besides choosing the ideal MB, it remains unclear whether nanocarrier‐encapsulated mRNA should be administered separately (co‐administration) or conjugated to MB (co‐formulation). Therefore, the impact of poly(n‐butyl cyanoacrylate) MB co‐administration with mRNA‐DOTAP/DOPE lipoplexes or their co‐formulation on the transfection of cancer cells in vitro and in vivo is analyzed. Sonotransfection improved mRNA delivery into 4T1 breast cancer cells in vitro with co‐administration being more efficient than co‐formulation. In vivo, the co‐administration sonotransfection approach also resulted in higher transfection efficiency and reached deeper into the tumor tissue. On the contrary, co‐formulation mainly promoted transfection of endothelial and perivascular cells. Furthermore, the co‐formulation approach is much more dependent on the US trigger, resulting in significantly lower off‐site transfection. Thus, the findings indicate that the choice of co‐administration or co‐formulation in sonotransfection should depend on the targeted cell population, tolerable off‐site transfection, and the therapeutic purpose.

## Graphical abstract

mRNA Sonotransfection of tumors: mRNA Lipoplexes are administered during focused ultrasound exposure either alone, with microbubble (MB) co‐administration, or coupled to the MB in a co‐formulation approach. The approaches result in significantly different transfection efficiency, transfected tumor tissue compartments, and unspecific off‐target transfection pattern (created with BioRender.com).

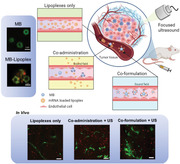

## 1 Introduction

The first human trial of mRNA therapy in 2009 demonstrated its suitability as a therapeutic tool and triggered great enthusiasm for genetic therapy, immunotherapy, and stem cell biomedicine.^[^ ^1^ ^]^ Yet, the effective delivery and transport of mRNA to the cytosol of target cells remains a major challenge as mRNA does not easily cross biological barriers. Furthermore, naked mRNA is vulnerable to immune recognition and extracellular RNase degradation.^[^ ^2^ ^]^

To address this, materials and delivery systems are being investigated to protect the mRNA from rapid degradation.^[^ ^3^ ^]^ In this regard, lipid nanoparticles are widely used carriers for gene delivery due to their low immunogenicity and ease of implementation.^[^ ^4^ ^]^ They often contain cationic lipids that can spontaneously interact with anionic mRNA through electrostatic interactions, forming lipoplexes.^[^ ^5^ ^]^ However, their gene transfection efficiency in vivo is limited, as they are not sufficiently stable and rapidly cleared through the reticuloendothelial system.^[^ ^6^ ^]^ To further improve their stability and transfection efficiency, helper lipids, such as dioleoyl phosphatidylethanolamine (DOPE), can be integrated to neutralize the cationic charge by their negatively charged phosphodiester and promote the endosomal escape of genes.^[^ ^7^ ^]^

Another challenge in achieving efficient transfection in vivo is the lack of target‐specific accumulation and transfection specificity. Local administration can work for superficial tissues like skin and muscle,^[^ ^8^ ^]^ but systemic intravenous injection is necessary for most deeper organs.^[^ ^9^ ^]^ Often lipoplexes must extravasate from blood vessels to reach their target cells, but structural barriers, like the dense extracellular matrix in tumors, hinder their penetration, leading to poor tumor accumulation.^[^ ^10^ ^]^ Additionally, the in vivo uptake of lipoplexes by endocytosis is typically inefficient. ^[^ ^11^ ^]^

To improve the mRNA accumulation in the tumor and to control the transfection spatially and temporally, sonotransfection with microbubbles (MB) is currently being evaluated. Focused ultrasound (US) can target specific areas or tissues while minimizing damage to healthy tissues. MB‐induced stable or inertial cavitation generates shear forces, leading to transient pore formation in cell membranes or opening of intercellular junctions,^[^ ^12^ ^]^ facilitating the penetration of nucleic acids through cell layers and into the target cells. This process, known as “sonoporation”, “sonopermeation” or “sonotransfection”, has been continuously optimized in vitro and in vivo.^[^ ^13^ ^]^ In this context, the chosen US parameters have a significant impact on the transfection efficiency and the unwanted cell viability loss, necessitating careful optimization to achieve therapeutic goals without harming healthy tissue.^[^ ^14^ ^]^

Furthermore, the success of sonoporation strongly depends on the type of MB, which differ in their oscillation and disintegration characteristics and require tailored US excitation schemes according to their physicochemical properties.^[^ ^15^ ^]^ Lipid‐coated MB, like Sonovue or Defintiy, commonly used in sonotransfection research, have a flexible shell leading to nonlinear oscillation (stable cavitation).^[^ ^16^ ^]^ However, their broad size distribution and potential fusion during US exposure complicate reproducibility and precise control in gene delivery experiments, as the MB size affects acoustic properties.^[^ ^17^ ^]^ In contrast, hard‐shell MB, characterized by a shell of proteins or polymers, tend to be more stable as their shell is more rigid, does show less or no buckling effects and better retains the gas. Furthermore, as hard‐shell MB do not coalesce with adjacent MB and as they better tolerate repeated differential centrifugation, they can be more easily produced with a narrower size distribution, which makes their acoustic properties more uniform. These MB show less stable cavitation in response to US but their destruction by inertial cavitation leads to a gas bubble that is ejected through a shell defect (loss of correlation) and then strongly oscillates.^[^ ^18^ ^]^ Both effects, stable and inertial cavitation lead to the transmission of US energies to the biological environment and are capable of opening endothelial and stromal barriers as well as cell membranes.^[^ ^19^ ^]^ Thus, they are supporting RNA delivery in the co‐administration approach. In the co‐formulation approach, however, where the RNA is transported via the MB, MB destruction is required. Here, polymer MB can be broken into smaller fragments that either penetrate neighbored cells or extravasate through leaky tumor vasculature and slowly release the payload in the tumor interstitium.^[^ ^20^ ^]^

Besides selecting the appropriate MB type, it is an unresolved question whether the mRNA‐containing carrier should be co‐administered or co‐formulated. While most in vivo studies have explored systemic co‐administration of free genetic material and MB,^[^ ^21^ ^]^ there is evidence that co‐formulation can enhance sonotransfection efficiency. For instance, Wang et al. found that the non‐covalent binding of plasmid DNA to cationic MB increases sonotransfection in vitro and in vivo compared to electroneutral MB.^[^ ^22^ ^]^ However, this coupling method is sensitive to pH changes in the medium (e.g., when getting in contact with plasma), leading to potential off‐target release of the genetic material.^[^ ^23^ ^]^ Alternatively, plasmid DNA can directly be coupled to the surface of MB.^[^ ^24^ ^]^ However, for structurally less stable single‐stranded mRNA this is problematic.

Nucleic acids can be incorporated in lipoplexes, which are then conjugated to the MB through specific binding techniques. For example, Lentacker et al. achieved higher transfection efficiencies using lipid‐coated MB coupled with lipoplexes through avidin and biotin bridging, surpassing free PEGylated lipoplexes or naked plasmid DNA.^[^ ^25^ ^]^ While these lipid‐coated MB‐based approaches show potential, the use of polymer MB for gene delivery would extend the capabilities for functionalization and nucleic acid loading and coupling.^[^ ^26^ ^]^ Furthermore, sonotransfection with mRNA has never been explored in vivo using such an approach.

Therefore, we explored the potential of poly(n‐butyl cyanoacrylate) (PBCA) MB, widely used in US molecular imaging and drug delivery for sonotransfection.^[^ ^27^ ^]^ For the first time, we systematically evaluated the effects of MB and mRNA lipoplex co‐administration and co‐formulation in vitro and in vivo using breast cancer‐bearing mice. Both sonotransfection approaches improved mRNA delivery in vitro and in vivo. However, we could identify significant differences in transfection efficiency, tissue compartment preference, and specificity to US triggering, the latter affecting off‐site transfection of other organs.

## 2 Result and Discussion

### 2.1 Generation of the In Vitro Sonotransfection Setup

**Figure 1:**
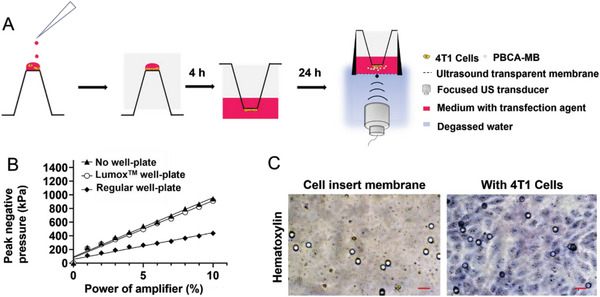
In vitro ultrasound setup. A) Schematic diagram of the inverted cell culture on cell inserts in Lumox^TM^ well‐plates. B) Focal acoustic pressures measured without a well‐plate, a Lumox^TM^ well‐plate, and a regular well‐plate, quantified by placing a needle hydrophone at the focus and adjusting the amplifier output to achieve an increase in percentage. Simple linear regression models were applied to plot the relation between the focal negative peak pressure (output variable in kPa; Y‐axis) and the amplifier output (input variable in %). The negative peak pressure linearly increases with the amplifier output and there is hardly a difference between the condition without and with the Lumox^TM^ well‐plate, while the plastic of the regular well‐plate absorbs and reflects most of the US. C) Hematoxylin staining of 4T1 cells growing on the insert membranes (scale bar = 10 µm).

To gain a deeper understanding of the interaction between MB and cells, in vitro experiments were conducted using Lumox well‐plates with a thin foil at the bottom (**Figure** **1**A), allowing US propagation from underneath with minimal attenuation. The acoustic pressure transmitted through the foil was measured with a hydrophone positioned at the US focus point for the three groups: no plate, regular well‐plate, and the Lumox well‐plate. Lumox well‐plates hardly absorbed US with a linear relationship between focal negative peak pressure and amplifier output (83 ± 5 kPa increase for each 1 % increase in amplifier output). These values were close to the no‐plate group (87 ± 3 kPa increase for each 1 % increase in amplifier output). In contrast, significant acoustic attenuation was found for regular well‐plates (only 39 ± 9 kPa increase for each 1 % increase in amplifier output, Figure 1B).

As MB float to the surface, cells were grown at the bottom side of filter inserts to enable direct contact with the MB.^[^ ^28^ ^]^ Confluence of 4T1 cells was confirmed by hematoxylin staining (Figure 1C).

### 2.2 Cell Viability and MB Response under In Vitro US Exposure

**Figure 2:**
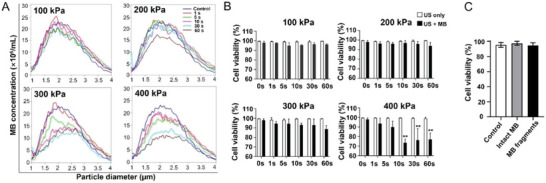
Optimization of in vitro sonotransfection parameters. A) Quantification of cell viability by FDA/PI double staining under different acoustic pressures and sonication times with or without MB (*p<0.05, **p<0.01; 0 s indicates no US). A significant decrease in cell viability is only found at 400 kPa in presence of MB at 10, 30, and 60 s sonication time. However, viability always remains over 70%. B) Change in MB numbers after exposure to different US pressures. MB numbers strongly decrease above 300 kPa dependent on the sonication time. C) Cell viability after incubation with intact MB and MB fragments using the XTT assay. Both intact MB and MB fragments do not affect 4T1 cell viability.

For efficient gene therapy, it is important that cell viability is not impaired by the intervention. Therefore, the effect of different US settings on cell viability was investigated. Careful control of acoustic parameters, especially the intensity and sonication time, can minimize risks associated with sonotransfection, such as overheating, or cell death caused by mechanical force. US alone did not affect cell viability between 100 to 400 kPa. Even with MB, cell viability was not significantly reduced up to 400 kPa and 1 s sonication time. However, at this high US energy at longer sonication times cell death increased significantly (Figure 2B). In addition, high pressures and long sonication times promoted MB disintegration (**Figure** **2**A, Table S1, Supporting Information). It is worth noting that the cell viability was not affected when cells were incubated with both MB and MB fragments without applying US (Figure 2C), which suggests that the changes in cell viability are not related to the materials directly but to the transmission of acoustic energies.

Although MB destruction is required for hard‐shell MB sonotransfection, the strong shear forces generated by MB rupture at high pressures may have increased the apoptosis rate in 4T1 breast cancer cells.^[^ ^29^ ^]^ In this context, Karshafian et al. showed that at high energies many cells fail to repair their membrane after sonoporation and die.^[^ ^30^ ^]^ However, it is noteworthy that even at the extreme condition of 400 kPa and 60 s sonication time, cell death never exceeded 26.4 ± 3.6 %, which is a number that was still considered acceptable in other gene therapy approaches.^[^ ^31^ ^]^

### 2.3 US with MB Enhances mRNA Delivery In Vitro

To optimize the US setting, lipofectamine 3000 was used, which is a commercially available delivery system and is considered a gold standard for in vitro transfection purposes.^[^ ^32^ ^]^ However, for the subsequent in vivo applications it was unfavorable due to its highly positive surface charge, which leads to a short blood circulation time, high offsite transfection, and significant toxicity. ^[^ ^33^ ^]^ Furthermore, it could not be functionalized and conjugated to MB. Therefore, lipoplexes were used for subsequent in vitro and in vivo experiments.

**Figure 3:**
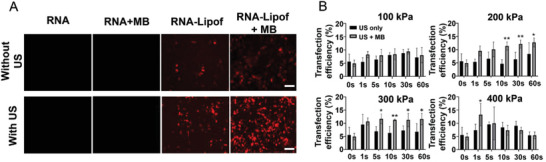
Results of the in vitro transfection of 4T1 cells using different sonotransfection parameters. A) Representative images of transfected 4T1 cells using mCherry mRNA alone, mRNA with MB, mRNA‐loaded lipofectamine^TM^ 3000 alone (RNA‐Lipof), and RNA‐Lipof with MB in the presence and absence of US (400 kPa; 1 s, scale bar = 100 µm). Without RNA‐Lipof, transfection of 4T1 cells is hardly visible even when delivered in presence of US with MB. The combination of US and MB significantly enhances the mRNA‐Lipof transfection in vitro. B) Quantification of transfection efficiencies using mRNA‐Lipof under different acoustic pressures and sonication times with and without MB (compared to US only, *p<0.05, **p<0.01).

With naked mRNA, even with US and MB, no transfection of 4T1 cells was detected (**Figure** **3**A). This is consistent with previous reports stating that naked mRNA gives low transfection efficiency.^[^ ^34^ ^]^ The transfection efficiency was calculated as the percentage of transfected cells in relation to the total number of cells. With lipofectamine 3000 a transfection efficiency of 4.9 ± 1.1 % was achieved in 4T1 cells (Figure 3A,B), which was below the manufacturer's optimal values (≈70 %). While the 4T1 cells showed satisfactory growth on the membrane of the filter inserts, the inverted culture might have led to reduced uptake by the cells.^[^ ^35^ ^]^ Besides this, the limited transfection efficiency could be due to differences in uptake between cell lines. Comparing US alone with US with MB at negative acoustic pressures of 200, 300, and 400 kPa significantly enhanced transfection efficiencies were found, which depended on the sonication time (Figure 3B). In detail, after 10 s sonication with 200 kPa significantly more cells were transfected in presence than in absence of MB. At 300 kPa a similar result was already obtained after 5 s (Figure 3B). Applying US pressures of 400 kPa for 1 s resulted in the best transfection efficiency (13.2 ± 4.2 %) without significantly decreasing cell viability in the US plus MB group. Interestingly, no differences compared to US alone were found for longer sonication times. The latter can be explained by the increasing loss in cell viability (Figure 2B), likely impacting the transfection efficiency.

From a translational perspective, it is preferable to keep the sonication times as short as possible, to minimize the risk of microhemorrhages and comply with the relatively short lifetime of MB in the blood. Therefore, 400 kPa and 1 s sonication time were used for all subsequent in vitro experiments.

### 2.4 Production and Characterization of Lipoplexes and MB

In vivo gene delivery experiments are often performed using DOTAP/DOPE lipoplexes, as they compensate for some of the limitations of lipofectamine 3000 and offer versatile options for customization.^[^ ^36^ ^]^ Their disadvantage is that transfection efficiency is often lower than for lipofectamine 3000, which was also true for our study (Figure S2, Supporting Information). However, transfection rates were still sufficient to perform our experiments and answer our scientific questions.

Co‐administration and co‐formulation approaches of lipoplexes and MB have shown promise in DNA delivery studies,^[^ ^25^, ^37^ ^]^ but their suitability for sonotransfection with mRNA remains unclear. Furthermore, previous experiments have not been performed using hard‐shell MB, and a detailed site‐to‐site comparison between the transfection pattern of co‐administration and co‐formulation approaches has not been done in vivo yet.

**Figure 4:**
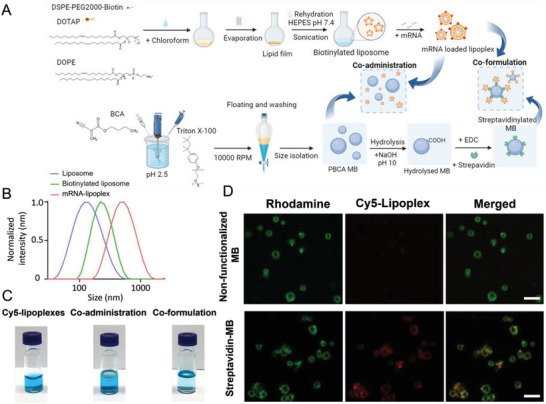
Synthesis and characterization of the lipoplexes, MB, and lipoplex‐MB conjugates. A) Scheme illustrating the synthesis of lipoplexes, MB, and lipoplex‐MB conjugates. B) Intensity‐based DLS results of the size distribution of DOTAP/DOPE liposomes, biotinylated DOTAP/DOPE liposomes, and mCherry mRNA‐loaded DOTAP/DOPE lipoplexes. The mCherry mRNA‐loaded DOTAP/DOPE lipoplexes are slightly larger than their unloaded counterparts. C) Formulations of Cy5‐lipoplexes only (left), Cy5‐lipoplexes mixed with MB (floated to the top, middle), and Cy5‐lipoplexes coupled to MB (right). D) Fluorescence images of MB and lipoplex‐conjugated MB. MB are pre‐dyed with rhodamine (in green), while the lipoplexes are loaded with Cyanine 5 (Cy5, in red). Scale bar = 5 µm.

To address this, we prepared cationic liposomes loaded with mRNA using a film‐hydration method (**Figure** **4**A). Successful mRNA incorporation was confirmed by the larger size of the DOTAP/DOPE mRNA‐loaded lipoplexes compared to the original liposomes and biotinylated liposomes (344.2 ± 13.1 nm versus 126.4 ± 0.9 nm and 179.4 ± 0.9 nm, Figure 4B). The increased size may arise from electrostatic interaction between cationic lipids and mRNA, likely leading to changes in the conformation and organization of the lipids.^[^ ^38^ ^]^ For the co‐formulation approach, DOTAP/DOPE lipoplexes were biotinylated with 5 % DSPE‐PEG2000‐biotin and mixed with streptavidinylated PBCA‐MB, which were synthesized as reported by Palmowski et al..^[^ ^39^ ^]^ Using the BCA protein assay, an amount of (9.5 ± 0.2) x 10^−7^ µg streptavidin per MB was found (Figure S4, Supporting Information). The Ribogreen assay showed 230 ± 22 ng mRNA encapsulated in 10^7^ lipoplex‐MB complexes. 10.9 ± 1.2 % of the added lipoplexes were bound to MB (Figure S5, Supporting Information). Furthermore, we loaded the core of the lipoplexes with Cy5 dye to visualize them in fluorescence microscopy. Optically, the co‐administration solution showed an MB cake as the top layer, with blue Cy5‐lipoplexes as subnatant. In contrast, the co‐formulated solution showed a clear subnatant and colored MB cake (Figure 4C). Furthermore, the successful co‐formulation was validated by confocal laser scanning microscopy showing the co‐localization of Cy5‐lipoplexes (red) and rhodamine‐labeled MB (green) (Figure 4D). We analyzed the stability of liposomes, biotinylated liposomes, streptavidin‐MB, and liposome‐MB complexes at 4 and 37 °C. Both liposomes and biotinylated liposomes were stable for more than 3 days. Furthermore, streptavidin‐MB were stable for over 24 h at 4 °C and 12 h at 37 °C. The liposome‐MB complexes remained stable for 10 h at 4 °C and 3 h at 37 °C (Figure S6, Supporting Information). However, the relatively short stability of the liposome‐MB complexes did not affect the experiments as they were freshly prepared and as the liposome‐MB are cleared from the blood within less than 1 h.^[^ ^39^ ^]^

### 2.5 Comparison of mRNA Transfection Efficiency for Co‐Administration and Co‐Formulation In Vitro and In Vivo

**Figure 5:**
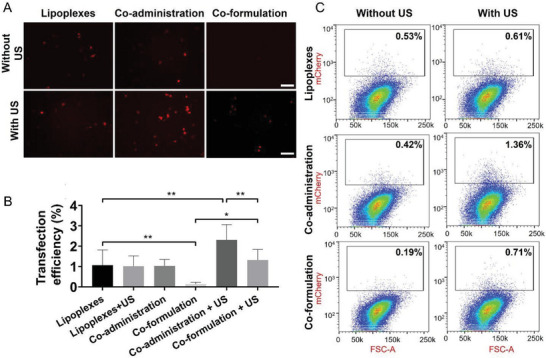
In vitro evaluation of the transfection efficiencies. A) Representative fluorescence microscopy images of 4T1 cells transfected with mCherry mRNA using lipoplexes only, co‐administration of lipoplexes and MB, and the co‐formulation approach with and without US (400 kPa; 1 second, 100×, scale bar = 100 µm). B) Quantification of transfection efficiency based on fluorescence analysis. The co‐administration approach with US results in the highest transfection efficiency, whereas the highest specificity is detected for the co‐formulation approach. *p<0.05, **p<0.01. C) Quantification of in vitro transfection efficiency of 4T1 cells using flow cytometry. Histogramms show the percentage of mCherry positive cells. The highest transfection efficiency is found for the co‐administration approach with US. Also in the co‐formulation approach, US increases the mRNA transfection efficiency.

In contrast to Abuhelal et al., who showed that the uptake of mRNA‐loaded lipoplexes can increase in the presence of US, ^[^ ^40^ ^]^ we did not find an US effect on the transfection efficiency in absence of MB (**Figure** **5**A,B).

However, both co‐administration and co‐formulation approaches significantly improved the mRNA transfection efficiency in the presence of US, which was confirmed by fluorescence microscopy (Figure 5A,B) and flow cytometry (Figure 5C). Furthermore, co‐administration resulted in a higher transfection efficiency than co‐formulation (Figure 5A,B). On the other hand, hardly any cells were transfected if the co‐formulated system was applied without US, similar to what was reported by Lentacker et al..^[^ ^25^ ^]^ This indicates that the co‐formulation approach requires the US trigger to disrupt MB and to release lipoplexes, making the transfection more specific and the efficiency more tunable.

**Figure 6:**
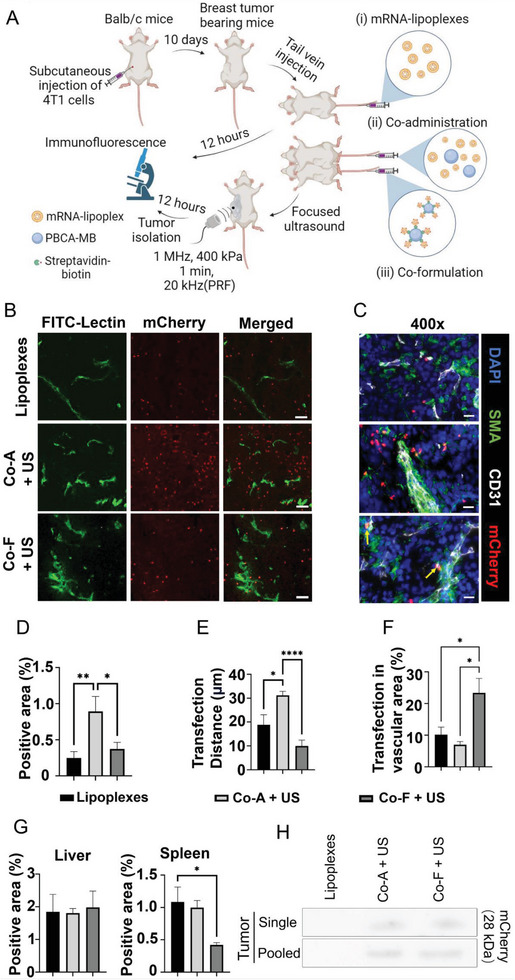
In vivo evaluation of transfection efficiencies. A) Scheme of the animal experiment (created with BioRender.com). B) Representative confocal microscopy images of tumor tissue transfected with mCherry mRNA (red) using lipoplexes only, co‐administration (Co‐A), and the co‐formulation (Co‐F) approach with and without US (200×, green = vessels, scale bar = 5 µm). C) Representative immunofluorescence images (400×) showing the subsets of endothelial cells (CD31, white) and smooth muscle cells (SMA, green) transfected with mCherry (red) (scale bar  =  20 µm). Transfected cells are found in larger distance from the vessels in the co‐administration approach, while preferably the (peri)vascular area is transfected by co‐formulation (indicated by yellow arrows). D, E, F) Quantification of the transfected tumor area, the distance of transfected cells from the nearest vessel, and the transfection efficiency of the (peri)vascular area, respectively. Sonotransfection with co‐administration achieved the highest transfected area and the highest transfection depth related to the nearest vessels. The co‐formulation with US resulted in a higher transfection of the (peri)vascular area than co‐administration with US or lipoplex only. G) Quantification of the unspecific liver and spleen transfection, respectively. The co‐formulation sonotransfection approach induced significantly less unspecific transfection of the spleen than the co‐administration approach. *p<0.05, **p<0.01, ****p<0.001. H) Immunoblots confirming the results of the fluorescence microscopy analysis shown in C‐F.

Subsequently, the transfection efficiencies of lipoplexes alone, co‐administration, and co‐formulation approaches were compared in 4T1 breast cancer‐bearing mice (**Figure** **6**A). Intravenous injection of streptavidin‐coated MB did not induce an inflammatory response in mice (Figure S10, Supporting Information). Lipoplex administration led to low tumor transfection efficiency despite the “enhanced permeability and retention” (EPR) effect.^[^ ^41^ ^]^ This is in line with the increasingly critical discussion about the heterogeneity of the EPR effect in different tumor models and its low expression in many human tumors.^[^ ^42^ ^]^ Therefore, specific manipulation of the involved biological barriers, e.g., by sonoporation, might often be necessary to prepare tissue for EPR‐based particle delivery.^[^ ^42^ ^]^

Consistent with our in vitro experiments, fluorescence microscopy indicated that sonotransfection significantly enhanced the transfection compared to lipoplexes alone. In this context, co‐administration was superior to co‐formulation (lipoplexes alone: 0.25 ± 0.1%; co‐administration: 0.90 ± 0.2% (increase by 260%), co‐formulation: 0.37 ± 0.1% (increase by 48%); Figure 6B,D). This finding was confirmed by immunoblot (Figure 6H). Furthermore, co‐administration resulted in significantly deeper tissue transfection than the other approaches (Figure 6B,E). The mean distance between the transfected cells and the nearest blood vessel was found to be 31.2 ± 1.6 µm with co‐administration plus US, 18.9 ± 4.1 µm with lipoplexes only, and 9.9 ± 2.5 µm with co‐formulation plus US (Figure 6E). The different sonotransfection strategies also influenced the cell types being transfected. Co‐formulation plus US resulted in a higher transfection of the vascular area (endothelial cells and adjacent pericytes and smooth muscle cells; 23.4 ± 4.6%) than lipoplexes alone (10.1 ± 2.4%) and co‐administration plus US (7.0 ± 1.7%, Figure 6B,6F), the latter transfecting a higher fraction of 4T1 cancer cells. Furthermore, by analyzing the cellular subsets in the vascular area (Figure 6C), we found that in the co‐administration approach 7.4 ± 3.0 % of the transfected cells were endothelial cells and 35.7 ± 4.7 % smooth muscle cells, while in the co‐formulation approach these were 56.8 ± 13.5 %, and 42.2 ± 8.9 %, respectively (Figure S11, Supporting Information).

Another important factor influencing the in vivo transfection efficiency is the duration of sonoporation‐mediated barrier opening, allowing the extravasation of lipoplexes, which can vary from minutes to hours.^[^ ^43^ ^]^ In the co‐administration approach circulating mRNA‐loaded lipoplexes can penetrate the vessel wall and the permeabilized cells for hours after the US treatment. In contrast, in the co‐formulation approach, mRNA‐loaded lipoplexes can only be released when MB disintegrate during US exposure. Thus, the time, in which mRNA‐loaded lipoplexes can extravasate is much shorter. This explains the less deep and predominantly (peri‐)vascular transfection.

Although the co‐administration approach reached considerably deep, it is noteworthy that cavitation effects can only be generated within blood vessels because MB cannot extravasate. In this regard, improved and more homogeneous transfection of cell layers that are distant from blood vessels may be achieved by using US‐responsive nanoscale systems (e.g., nanobubbles or phase‐change nanodroplets), which can effectively extravasate and accumulate in the tumor interstitium.^[^ ^44^ ^]^

Next, we examined the off‐target transfection of liver and spleen as these are essential organs for the clearance and metabolism of xenobiotics, including nucleic acids and gene carriers. Unwanted transfection in these organs can potentially compromise the safety and efficiency of this gene delivery system. No significant differences in the transfection of liver cells were found between the lipoplex‐only (1.8 ± 0.5 %), co‐administration plus US (1.8 ± 0.3 %), and co‐formulation plus US approaches (2.0 ± 0.5 %; Figure 6G). In the spleen, off‐site transfection in the co‐administration based sonotransfection group (1.0 ± 0.1 %) was not significantly different from the lipoplex‐only group (1.1 ± 0.2 %), while it was less than half in the co‐formulation sonotransfection group (0.4 ± 0.0 %; Figure 6G), suggesting that the co‐formulation reduces unwanted accumulation in the spleen. Therefore, the co‐formulation approach can be beneficial in cases where off‐site transfection needs to be minimized.

Contrary to our findings, previous studies showed that the highest transfection efficiency of plasmid DNA in vitro can be achieved when the lipoplexes are attached to the lipid‐coated MB (equivalent to the co‐formulation approach) and are not just co‐administered with MB.^[^ ^45^ ^]^ There are several explanations for the divergent outcomes: We used mRNA instead of plasmid DNA, which is much less stable than mRNA. When MB disrupt, part of the nucleic acids may be released from the lipoplexes. Free plasmid DNA may still benefit from the opened cell membrane, while free mRNA will not transfect cells efficiently (as shown by our results in Figure 3A). Furthermore, different MB were used that have divergent destruction characteristics. Lipid‐coated MB disintegrate into many small bubbles that may carry the lipoplexes into the cells. In contrast, the PBCA‐MB fall apart into multiple polymer fragments with sizes of up to 300 nm.^[^ ^46^ ^]^ We assume that larger fragments that still carry lipoplexes will mostly remain outside of the cells, thus reducing transfection efficiency. Choosing a sonoresponsive coupling technique between MB and lipoplexes, e.g. via hydrogen bonds or finetuning the shell polymer properties to disintegrate into smaller fragments could solve this issue.^[^ ^47^ ^]^

The combination of PBCA‐MB and US successfully improved mRNA delivery. A single treatment with co‐administration transfected ≈1% of the tumor cells. There is no doubt that for many RNA‐based tumor treatments higher transfection rates might be required. These could easily be achieved by increasing the mRNA dosage, repeated treatments, or optimizing the timing of sonotransfection. Especially repeated sonotransfection could further enhance the efficiency, as multiple treatments have been reported to yield higher transfection without significant cytotoxicity. ^[^ ^48^ ^]^ Furthermore, there is also room for refinements in the ultrasound settings, the MB and the RNA carriers. However, it is noteworthy that in this study the aim was to compare free lipoplex administration with sonotransfection after co‐formulation and co‐administration, and thus, the relation of the transfection efficiencies between these groups is the most important readout. Here a substantial improvement could be noted after sonotransfection. Besides this, the timing of US application is crucial. In this regard, lipoplexes and MB were mostly injected simultaneously while applying US.^[^ ^49^ ^]^ However, cationic lipoplexes have a longer blood half‐life (≈36 h) compared to MB (5–10 min).^[^ ^50^ ^]^ Thus, it would be interesting to investigate whether letting lipoplexes accumulate in the tumor prior to sonotransfection is beneficial for gene delivery and how it changes the transfection pattern within the tissue. In these settings, the accumulation of fluorescently labeled lipoplexes in tumor tissue could be controlled by real‐time imaging. Real‐time imaging of mRNA expression may not be indicated, as it takes several hours until proteins are translated (Figure S3, Supporting Information). However, its longitudinal non‐invasive monitoring over hours and days is interesting to assess the onset and decline of protein expression, which is, e.g., important for therapeutic applications, where several injections are required. In this regard, bioluminescence imaging using luciferase mRNA would be an interesting alternative for further investigation due to its low background signal in vivo and therefore better detectability.

## 3 Conclusion and Outlook

We demonstrate the suitability of PBCA‐MB for enhancing mRNA sonotransfection. In vitro but also in mice with breast tumors, the different delivery approaches resulted in distinct transfection patterns. On the one hand, the co‐formulation approach allowed for a highly trigger‐dependent but vessel‐selective transfection, which, e.g., is valuable for treating cardiovascular diseases like atherosclerosis, inhibiting tumor angiogenesis, or promoting immune cell extravasation. On the other hand, the co‐administration resulted in higher efficiency and deeper tissue transfection, which, for example, is required to target desmoplastic tumor stroma or the cancer cells themselves. Furthermore, the co‐formulation approach reduced the unspecific off‐site transfection in mice. This can be therapeutically important as, for some therapeutic genes, off‐site transfection needs to be strongly avoided, while others demand maximal transfection for optimal therapeutic outcomes. Consequently, the choice between co‐administration and co‐formulation should be thoroughly considered based on the specific target and objective when delivering nucleic acids. In this context, it needs to be considered that repeated sonotransfection interventions may be required to achieve the desired therapeutic outcome, which, however, goes along with further risks of side effects. Therefore, improvements to the systems, e.g., by using targeted or differently shaped MB and better (e.g., more stable) mRNA carriers should also be the focus of future research.

## 4 Experimental Section

### Generation of the In Vitro Sonotransfection Setup

The Lumox 24 well‐plate (Sarstedt AG & Co. KG, Nümbrecht, Germany) containing the cell inserts with the 4T1 cells was exposed to a focused US system, which comprised a waveform generator with an integrated oscilloscope function (Model SDS1202X‐E, Siglent.eu, Helmond, Netherlands), a second waveform generator (Model 33622A, Keysight Technologies, Böblingen, Germany), a radiofrequency (RF) broadband power amplifier (Model AG1021, T&C Power Conversion, Rochester, NY, USA), and a waterproof 1 MHz focused immersion transducer (Model V308‐SU, Olympus Europa SE & Co. KG, Hamburg, Germany) in a water tank. The transducer's surface was 2.5 cm away from the bottom of the 24 well‐plates, which allowed the adjustment of the focal point to the position of the samples. To compare the acoustic property between the regular well‐plate (Corning, Taufkirchen, Germany) and the Lumox 24 well‐plate, the focal acoustic pressure was measured using a 0.2 mm needle hydrophone positioning at the focus within the wells (Precision Acoustics Ltd., Dorchester, UK).

### Cell Culture

The murine breast cancer cell line 4T1 (American Type Culture Collection, Virginia, USA) was cultured in Dulbecco's modified Eagle's medium (DMEM, Gibco, Germany) containing 10 % fetal bovine serum (Gibco, Germany) and 1 % penicillin/streptomycin (Gibco, Germany). The cells were kept in a humidified atmosphere containing 5 % CO_2_ and passaged at 70%–80 % confluency. Before the experiments, cells were transferred to polycarbonate cell culture inserts (Corning, Darmstadt, Germany) in 24 well‐plates (ThermoFisher Scientific, Schwerte, Germany) to generate a setup for US exposure, in which MB are close to the cells. For this purpose, the cell inserts were placed upside‐down, and 1×10^6^ 4T1 cells were seeded on the underside of the filter membranes and incubated for at least 4 h. Then, 1 mL medium was added to each well, and the cell inserts were turned back into the normal position, followed by further incubation for 24 h to reach confluence.

### Synthesis and Surface Functionalization of PBCA‐MB

PBCA‐MB were synthesized by anionic‐emulsion polymerization as described previously.^[^ ^51^ ^]^ Shortly, 3 mL of n‐butyl cyanoacrylate (BCA, Special Polymers, Sofia, Bulgaria) was added dropwise to 300 mL aqueous solution containing 1 % of Triton X‐100 at pH 2.5. This mixture was emulsified using an Ultra‐Turrax T‐50 basic device (IKA Werke, Staufen, Germany) at 10000 RPM for 1 h at room temperature. The resulting solution was centrifuged at 500 RPM for 10 min and washed with 0.02% (v/v %) aqueous solution of Triton X‐100 (pH 7, Sigma–Aldrich, Munich, Germany) until the subnatant became transparent. We used carbodiimide crosslinker chemistry to functionalize the MB‐shell for the co‐formulation approach. The butyl‐ester groups of the PBCA sidechains were incompletely hydrolyzed by adding NaOH (0.1 M, AppliChem, Darmstadt, Germany) dropwise to increase the pH value to 10. The suspension was then stirred in a round bottom flask at 400 RPM for 15 min. MB size and concentration were determined using a Multisizer 4e (Beckman Coulter, California, USA). 10^9^ hydrolyzed MB were activated by 15 mg EDC (N‐(3‐dimethylaminopropyl)‐N‐ethylcarbodiimide hydrochloride, Sigma–Aldrich, Munich, Germany) at pH 4.5, followed by the addition of streptavidin (300 µg, ChemImpex, Wood Dale, USA) to the MB suspension. The mixture was stirred at 4 °C overnight. The functionalized MB were purified by multiple washing steps using 0.02 % Triton X‐100 to remove the impurities. Streptavidin‐MB were stored in HEPES buffer containing 0.01% Triton X‐100. These PBCA‐MB are known to be stable in suspension for over 20 weeks.^[^ ^52^ ^]^

### Preparation and Characterization of Biotinylated Cationic Liposomes and Lipoplexes

The following phospholipids were obtained from Avanti Polar Lipid (Birmingham, Alabama, USA): N‐(1‐(2,3‐dioleoyloxy)propyl)‐ N,N,N‐trimethylammonium chloride (DOTAP), dioleoyl phosphatidyl ethanolamine (DOPE), DSPE–PEG2000–biotin. The lipid powder, consisting of DOTAP and DOPE with a 1:1 weight ratio and 5 % (w/w) DSPE–PEG–biotin was fully dissolved in chloroform. The solvent was rotationally evaporated under a vacuum for 45 min to generate lipid films (200 rpm, 45 °C). The dried lipid films were rehydrated with 20 mM 4‐(2‐hydroxyethyl)−1‐piperazineethanesulfonic acid (HEPES, Sigma–Aldrich, Munich, Germany) in RNase‐free water (Invitrogen, Massachusetts, USA) and then incubated at 4 °C overnight to allow cationic liposome formation. Liposome solutions were vortexed for 1 min and then sonicated at 40 °C for 5 min to form small unilamellar vesicles. The obtained stock solution of biotinylated liposomes was stored at 4 °C until use. Lipoplexes were formed by mixing the DOTAP/DOPE cationic liposomes with mCherry mRNA (Miltenyi Biotec, Bergisch Gladbach, Germany) at a negative‐to‐positive charge ratio of 1:1 in RNase‐free HEPES buffer (Invitrogen, Paisley, UK). The mRNA was first diluted in HEPES buffer to a concentration of 0.5 mg mL^−1^. Then, the mRNA solution was added to an equal volume of cationic liposome suspension (2.2 mg/mL of lipid concentration). The resulting mixture was incubated for 30 min at room temperature. The lipoplexes and lipoplex‐conjugated MB were stored in HEPES buffer. The sizes of both, the cationic liposomes and the mRNA‐loaded lipoplexes, were determined by dynamic light scattering (DLS, Zetasizer Nano ZS, Malvern, UK) at 25 °C. Data were analyzed using software supplied with the Zetasizer. Results represent the average of at least three measurements per run, with more than 10 runs performed per sample.

### Assessment of mRNA Encapsulation Efficiency

The amount of mRNA encapsulated inside the lipoplexes was determined using the Quant‐iT RiboGreen RNA Assay Kit (Thermo Fischer Scientific, Waltham, USA). Free, unencapsulated mRNA was removed by ultrafiltration with Vivaspin 500 (300 kDa, Sartorius, Goettingen, Germany) at 5000 RPM for 5 min. As the Ribogreen reagent does not penetrate liposomal membranes, 0.5 % Triton X‐100 was applied to disrupt the membranes of the lipoplexes. Measurements were performed using the TECAN Infinite M200 Pro microplate reader (TECAN group Ltd., Maennedorf, Switzerland) with excitation and emission at 480 nm and 520 nm, respectively. The mRNA concentrations of the samples were calculated based on standard curves of ribosomal RNA (ranging from 0 to 1000 ng).

### Attachment of Biotinylated Lipoplexes to Streptavidin‐Conjugated PBCA‐MB

10^9^ streptavidin‐functionalized PBCA‐MB were added to 2.5 µL of the biotinylated lipoplex solution, and the suspension was stirred at 400 RPM at room temperature for 10 min. Additional washing steps were performed to remove the unbound biotinylated lipoplexes.

The mRNA content in the lipoplex‐MB complexes was determined as described above. The loading efficiency (%) of lipoplex to MB was calculated by dividing the concentration of mRNA measured in lipoplex‐MB by the concentration of mRNA measured in the initially added lipoplexes.

For further characterization of the binding of lipoplexes to MB, lipoplexes were fluorescently labeled. Here, the thin lipid films were rehydrated with 1 mg mL^−1^ Cyanine‐5‐amine (Cy5, Lumiprobe, Hannover, Germany) in HEPES buffer. Unencapsulated Cy5 was removed by Disposable PD 10 Desalting Columns (Sigma–Aldrich, Darmstadt, Germany). Additionally, the MB shell was labeled with rhodamine: 1 mg of rhodamine‐B (Merck, Darmstadt, Germany) was added to 10 mL of the pre‐formed PBCA‐MB suspension under continuous stirring at 400 RPM for 4 h. The resulting solution was purified by washing in a 0.02 % (w/w) Triton X‐100 solution (pH 7) until the subnatant was free of residual dye. 10 µL of the sample was transferred to a glass slide and covered with a coverslip, followed by fluorescence microscopy. The Cy5‐labeled lipoplex and rhodamine‐B‐loaded MB were only used for the characterization. For all in vitro and in vivo co‐administration and co‐formulation experiments non‐fluorescent particles were used.

### Cell Experiments with US

Sonication was performed at 1 MHz with a pulse repetition frequency of 20 kHz and a duty cycle of 2 %. To optimize the acoustic pressure and sonication time, the cell viability, intact MB numbers, and the in vitro transfection efficiency of 4T1 cells with or without PBCA‐MB were evaluated at acoustic pressures of 100, 200, 300, and 400 kPa (mechanical index between 0.1 – 0.4) and 1, 5, 10, 30, and 60 s of sonication, respectively.

### Cell Viability Assay

Cell viability was assessed using a rapid double‐staining method of fluorescein diacetate (FDA, Sigma Aldrich, Darmstadt, Germany) and propidium iodide (PI, Sigma Aldrich, Darmstadt, Germany) after sonication (100–400 kPa, 1–60 s) with or without MB (10^9^/mL). Briefly, the cell inserts with 4T1 cells were washed 3 times with PBS after US exposure. The staining solution was prepared by mixing 8 µL FDA stock solution (5 mg mL^−1^ in acetone), 50 µL PI stock solution (2 mg mL^−1^ in PBS), and 10 µL Hoechst stock solution (200 mg mL^−1^ in PBS) in 5 mL PBS. 200 µL staining solution was added per well, and the cell inserts were incubated at room temperature for 10 min. Then, cell inserts were gently washed with PBS three times to remove the free dye. The basolateral membrane of the inserts was cut off for fluorescence microscopy. Five random ROIs per slide were captured by fluorescence microscopy (Axio Imager M2, Carl Zeiss, Oberkochen, Germany). The cell viability was calculated using the following formula: Cell viability (%) = [(number of green cells (FDA)/(total number of cells (Hoechst)] ×100 %.

Furthermore, the cell viability after incubation with MB and MB fragments was determined by colorimetric XTT assay according to the manufacturer's protocol (Thermo Fisher Scientific, Schwerte, Germany). To generate fragments, MB were destroyed using an ultrasonic cleaner (UCS 200TH, VWR, Darmstadt, Germany) by sonicating 1 mL solution containing 10^9^ MB for 1 min at 60 W. For the XTT assay, 5×10^3^ cells were plated in a 96‐well plate (100 µl medium per well) and mixed with 20 µL MB or MB fragments. After 4 h, 50 µL XTT reagent was added, and the cells were incubated for another 4 h for chromogen development. The absorbance was measured at 450 nm, with 630 nm as reference wavelength.

### In Vitro Transfection Experiments

Sonotransfection of 4T1 cells was performed in the following groups in the presence or absence of US (100–400 kPa, 1–60 s sonication time): free mRNA alone, free mRNA plus MB, mRNA loaded lipofectamine alone, and mRNA loaded lipofectamine plus MB. The mCherry mRNA was used at a concentration of 0.5 µg well^−1^ unless specified otherwise. The Lipofectamine 3000 Reagent (ThermoFisher Scientific, Schwerte, Germany), a commercial cationic lipid, was applied according to the manufacturer's protocol. For the groups with MB, 2×10^7^ PBCA‐MB were added to each well. The plate was sealed with parafilm, followed by US exposure. Then, the Lumox well‐plates were placed back in the cell culture incubator for 24 h, subsequently incubated with Hoechst 33342 solution (200 mg mL^−1^ in PBS, Sigma–Aldrich, Darmstadt, Germany), and washed with phosphate‐buffered saline (PBS, ThermoFisher Scientific Schwerte, Germany). Finally, the membranes were cut off by a surgical blade and mounted on glass slides.

DOTAP/DOPE was employed as a cationic genetic carrier in the following experiments to compare the mRNA delivery by co‐administration and co‐formulation. Transfection was performed in the following groups: lipoplex only, co‐formulation (lipoplexes‐MB complex), and co‐administration (lipoplexes mixed with MB) with and without US. Lipoplexes and MB were suspended in Opti‐MEM medium (ThermoFisher Scientific, Schwerte, Germany). Thirty min before the transfection experiment, the cell culture medium was removed, and cells were washed with PBS. Then, 200 µL of Opti‐MEM medium was added to each well of the Lumox well‐plate. 50 µL transfection solution (lipoplex only, co‐administration, or co‐formulation) was added to each well. Subsequently, the plates were exposed to US and incubated for 4 h. An additional 200 µL of medium containing 20 % FBS was added to each well to achieve a final serum concentration of 10 %, and plates were incubated for 24 h. The cellular nuclei were stained with Hoechst solution, and the transfection efficiency was assessed by fluorescence microscopy, randomly choosing 5 regions of interest (ROI). The CellProfiler 4.2.1 software (Cimini Lab, Broad Institute of MIT and Harvard, USA) was used to count transfected cells.^[^ ^53^ ^]^ The transfection efficiency was calculated using the following formula: Transfection efficiency (%) = [(number of red cells (expressing mCherry) / (total number of cells (Hoechst))]×100 %. Transfection efficiency was also determined by flow cytometry (BD Canto II). For this purpose, 4T1 cells were stained with DAPI 24 h after transfection, washed, and resuspended in PBS buffer. Flow cytometry was performed on a BD FACSCanto II (BD Biosciences). 1×10^5^ cells were measured. The results were analyzed by FlowJo v.10.0 (BD Life Sciences).

### In Vivo Experiments on Tumor‐Bearing Mice

All animal experiments were approved by the German State Office for Nature, Environment and Consumer Protection (LANUV) North Rhine‐Westphalia. 10–12 week old female Balb/c mice (Janvier Labs, Le Genest‐Saint‐Isle, France) were housed on spruce granulate bedding (Lignocel, JRS, Germany) in groups of 3–5 animals in type II long individual ventilated cages (Tecniplast, Germany) under specific pathogen‐free conditions. Husbandry rooms were temperature (20–24 °C) and humidity (45%–65 %) controlled. Water and standard pellets for laboratory mice (Sniff GmbH, Soest, Germany) were offered ad libitum. Group‐housed animals were assigned individual earmarks for identification. 4T1‐murine breast cancers were induced by subcutaneous injection of 4×10^4^ 4T1 cells. Tumors were allowed to grow to a maximum diameter of 5 mm before experimentation. For the mRNA sonotransfection experiments, mice were randomly assigned to the following groups: Lipoplex (n = 6), co‐administration + US (n = 6), and co‐formulation + US (n = 6). Mice were anesthetized with isoflurane and placed in prone position on a 37 °C heating pad. US gel was placed on the tumor to fill the gap between the skin and the transducer (Figure S7, Supporting Information). Sonoporation parameters were as follows: 1 MHz central frequency, 400 kPa peak negative acoustic pressure, and 20 kHz pulse repetition frequency, with a total sonication time of 60 s. The transfection suspensions (co‐formulation: 2×10^10^ MB kg^−1^ in 50 µL 0.9 % saline solution + 20 µl HEPES buffer; co‐administration: 2×10^10^ MB kg^−1^ in 50 µL 0.9 % saline solution + 2.12 mg k^−1^ g lipoplexes in 20 µL HEPES buffer) were injected intravenously through the tail vein (equal to 0.5 mg k^−1^ g mRNA each injection). The mice were injected with FITC‐lectin (Vector Laboratories Inc, Burlingame, California, USA) 12 h after treatment and euthanized by cervical dislocation after 15 min. Tumors of the mice were removed and snap‐frozen for further histological analysis. The livers and spleens of the mice were excised to investigate off‐target transfection.

### Histological Analysis

Immunofluorescence stainings were performed on 8 µm‐thick cryosections. Tumor, liver, and spleen sections were fixed with 80 % methanol and ice‐cold acetone for 5 and 2 min, respectively. After fixation, sections were washed 3 times with PBS and incubated with the anti‐mCherry antibody DyLight 680 (Novus Biologicals, Wiesbaden Nordenstadt, Germany) at a dilution of 1:100 overnight at 4 °C to detect the undenatured and denatured form of mCherry (Figure S8, Supporting Information). The next day, samples were washed thrice using PBS, air dried, and glass slides were mounted with coverslips using Mowiol 4–88 mounting solution. The specific expression of mCherry was analyzed by confocal laser fluorescent microscopy (Zeiss, Gottingen, Germany). The images were captured at 20x magnification.

To assign mRNA transfection to cell subsets, sections were incubated with rat monoclonal CD31 (1:20, Dianova, Hamburg, Germany) and mouse monoclonal anti‐alpha smooth muscle actin (SMA, 1:100, Progen, Heidelberg, Germany) primary antibodies at 4 °C overnight. Then, cyanine 3‐conjugated donkey anti‐rat IgG (H+L) (1:500, Dianova, Hamburg, Germany) and Alexa Fluor 488 conjugated goat anti mouse IgG (H+L) secondary antibodies (1:200, Dianova, Hamburg, Germany) were applied for 1 h at room temperature. Following this, sections were incubated in 4′,6‐diamidino‐2‐phenylindole (DAPI, Thermo Fisher Scientific, Schwerte, Germany) to stain and visualize the nuclei. ImageJ (National Institutes of Health, Bethesda, Maryland, USA) was used to analyze the tissue sections.^[^ ^54^ ^]^ The percentage of the transfected area was measured by calculating the percent positive area (in red). The distance between transfected cells and the closest vessel was determined by drawing a vertical line to the nearest vessel wall (stained in green). Values greater than 50 µm were excluded as the association to a specific vessel could not be ensured anymore (Figure S9, Supporting Information). Furthermore, to measure the transfection proportion, the vascular area (green‐positive area) was superimposed on the red‐positive area (transfected cells) by the software. The transfected vascular area (%) was calculated by dividing the co‐localized area by the total green‐positive area.

### Immunoblot

The expression of mCherry in the tumor tissue was evaluated. For this purpose, tumors were snap‐frozen and homogenized in a liquid nitrogen‐cooled tissue grinder. Subsequently, the resulting samples were incubated in RIPA buffer containing a protease inhibitor cocktail (Halt, Thermo Fisher Scientific, Schwerte, Germany) and Benzonase nuclease (Merck Millipore, Darmstadt, Germany) for 30 min. The total protein content of lysates was determined using the Pierce BCA protein assay kit (ThermoFisher Scientific). 30 µg of total protein per well were separated on a 4%–12 % polyacrylamide gel (Invitrogen) and blotted onto a nitrocellulose membrane using the Power Blotter System (Thermo Fisher Scientific). Membranes were blocked with 5 % non‐fat milk in TBS‐T for 60 min and hybridized with anti‐mCherry (1:800; Novus Biologicals, CO, USA) and anti‐vinculin (2 µg mL^−1^; R&D Systems, Minneapolis, MN, USA) primary antibodies overnight (4°C). After washing, membranes were incubated with an HRP‐conjugated secondary antibody at room temperature for 1 h. Finally, chemoluminescence signals were visualized on a luminescent image analyzer ImageQuant LAS 4000 (GE Healthcare, Chicago, IL, USA).

### Statistical Analysis

All data were analyzed with GraphPad prism 9.0 (San Diego, California, USA). Data are expressed as mean ± standard deviation. The one‐way analysis of variance (ANOVA) test was used for multiple‐group comparisons in combination with Tukey's honestly significant difference test. Differences were assumed statistically significant at p < 0.05.

## Conflict of Interest

The authors declare no conflict of interest.

## Supporting information

Supporting Information

## Data Availability

The data that support the findings of this study are available from the corresponding author upon reasonable request.
