# Supplementary material for: mRNA Sonotransfection of Tumors with Polymeric Microbubbles: Co‐Formulation versus Co‐Administration
Source: Adv Sci (Weinh). 2024 Feb 11;11(15):2306139. doi: 10.1002/advs.202306139 (PMC11022722; doi:10.1002/advs.202306139)
Supplement: Supplementary file 1 — Supporting Information [file ADVS-11-2306139-s001.pdf]

ADVANCED SCIENCE
Open Access

Supporting Information

for *Adv. Sci.*, DOI 10.1002/advs.202306139

mRNA Sonotransfection of Tumors with Polymeric Microbubbles: Co-Formulation versus Co-Administration

*Junlin Chen, Bi Wang, Yuchen Wang, Harald Radermacher, Jinwei Qi, Jeffrey Momoh, Twan Lammers, Yang Shi, Anne Rix and Fabian Kiessling**

Supporting Information

mRNA Sonotransfection of Tumors with Polymeric Microbubbles: Co-formulation vs. Co-administration

*Junlin Chen[1], Bi Wang[1], Yuchen Wang[1], Harald Radermacher[1], Jinwei Qi[1], Jeffrey Momoh[1], Twan Lammers[1], Yang Shi[1], Anne Rix[1#], Fabian Kiessling[1#]**

In vivo inflammatory response to streptavidin-coated MB: Eight female Balb/cAnNrJ mice aged 10-12 weeks (Janvier) were randomly assigned using Excel random numbers to receive either uncoated PBCA-MB or Streptavidin-coated PBCA MB (Strep MB). Housing conditions were like those described in the main text. After the acclimatization phase of one week, approximately 100 µl of blood was taken retrobulbar from the animals under inhalation anesthesia to obtain an individual baseline blood count for each animal. All blood analyses were performed from a person blinded to the treatment groups. One week after the first blood draw, the animals were administered the respective type of MB in a concentration of 2×10^9 MB/ml in a volume of 50 µl of 0.9% NaCl via a tail vein catheter. Subsequently, blood was taken retrobulbar from the animals to detect acute changes in the blood. A final blood sample was taken two days after MB injection to determine various blood parameters. The animals were euthanized by cervical dislocation, and gross necropsy was performed to assess changes in the liver, kidneys, and spleen weights.

Quantification of Streptavidin on MB: Streptavidin-conjugated MB were prepared as described in the Experimental section (page 24, last paragraph): 10^9/mL MB were destroyed by applying an ultrasonic cleaner for 1 minute at 60 W. The Pierce™ BCA Protein Assay was performed according to the manufacturer's instructions (Thermo Fisher Scientific, Schwerte, Germany). Briefly, bovine serum albumin (BSA) standards were prepared with concentrations ranging from 0 to 2 mg/mL. Aliquots of the protein samples (25 µL) or the standards were mixed with the BCA working reagent (200 µL) in a 96-well plate. The plate was incubated at 37 °C for 30 min to allow the formation of the purple-colored complex. After the incubation, the absorbance of the samples was measured at 562 nm using a microplate reader (Tecan, Maennedorf, Switzerland). A standard curve was generated using the BSA standards, and the protein concentrations of the samples were calculated based on the standard curve.

Table S1. MB concentrations (*10^9/mL) after exposure to different acoustic pressures and sonication times.

Acoustic pressure (kPa)	Sonication time (s)					Control
	1	5	10	30	60	
100	2.07 ± 0.24	2.12 ± 0.16	2.09 ± 0.16	2.02 ± 0.18	1.97 ± 0.32	2.15 ± 0.09
200	2.03 ± 0.49	2.02 ± 0.34	1.87 ± 0.13	1.70 ± 0.35	1.62 ± 0.39	
300	1.96 ± 0.06	1.96 ± 0.10	1.75 ± 0.39	1.52 ± 0.08	1.46 ± 0.42	
400	1.86 ± 0.12	1.86 ± 0.20	1.68 ± 0.18	1.42 ± 0.32	1.32 ± 0.16	

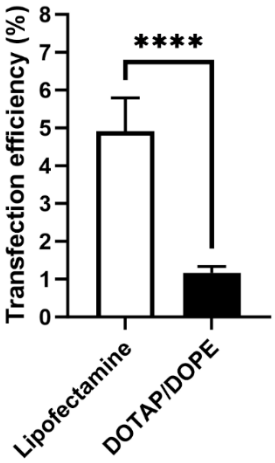

Figure S2. Comparison of the transfection efficiency in 4T1 cells using Lipofectamine® 3000 and DOTAP/DOPE lipoplexes. DOTAP/DOPE lipoplexes are approximately 75% less efficient than Lipofectamine® 3000.

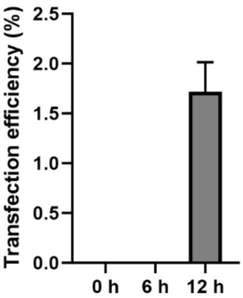

Figure S3. The percentage of mCherry expressing 4T1 cells 0, 6, and 12 h after transfection with mCherry mRNA loaded DOTAP/DOPE lipoplexes. mCherry is detectable after 12 h.

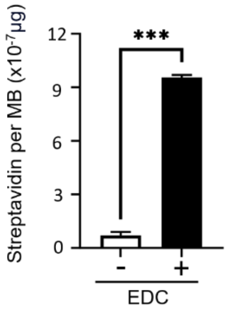

Figure S4. The quantification of streptavidin on MB. The addition of EDC significantly increases streptavidin binding. ***p < 0.001.

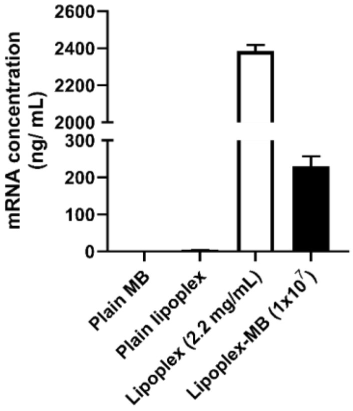

Figure S5. Quantification of mRNA contents in suspensions of plain MB, plain liposomes, mRNA-lipoplexes, and lipoplex-MB, as determined by the Ribogreen assay.

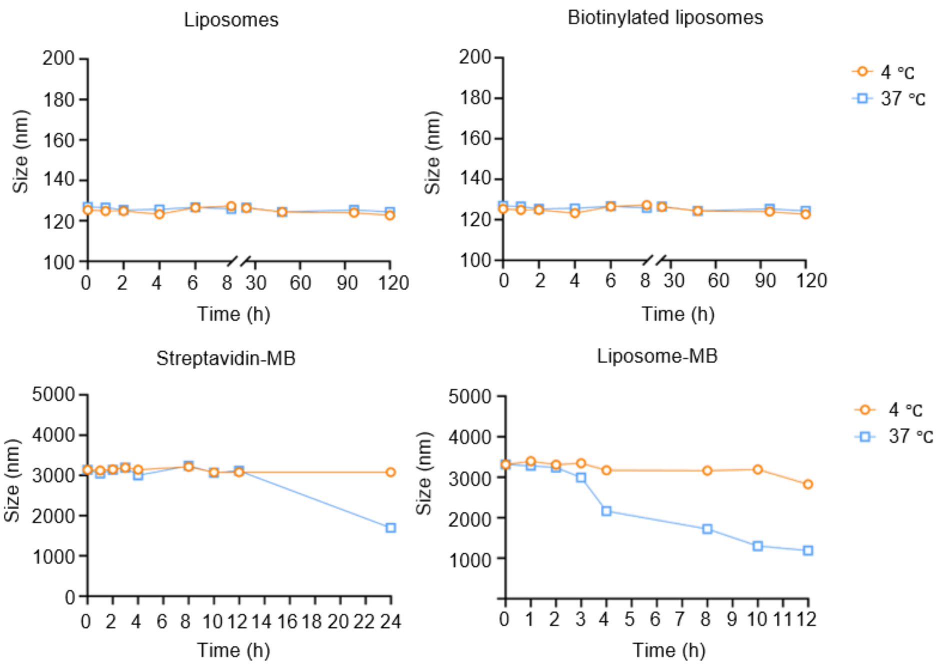

Figure S6. Sizes of liposomes, MB, and liposome-MB complexes at 4 °C and 37 °C over time. Liposomes and biotinylated liposomes are stable for more than 3 days at 4 °C and 37 °C as indicated by a stable size. At 4°C, streptavidin-MB are stable for more than 24 hours. At 37 °C, their size starts to decrease after 12 hours. Liposome-MB complexes remain stable for 10 hours at 4 °C. When exposed to 37 °C, their size starts to decrease after 3 hours, indicating that liposomes detach from the MB.

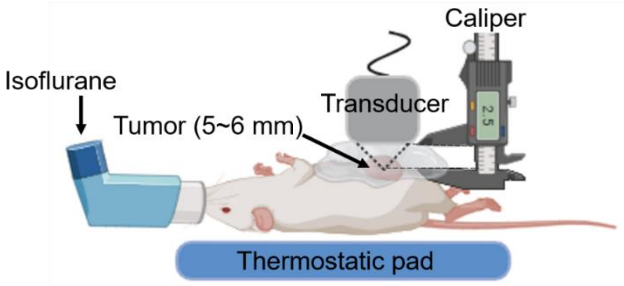

Figure S7. Sonotransfection of breast cancer-bearing mice. Mice were anesthetized with isoflurane and placed in prone position on a 37 °C heating pad. The transducer was kept 2.5 cm away from the center of the tumor using a caliper. US gel was placed on the tumor to fill the gap between the skin and the transducer.

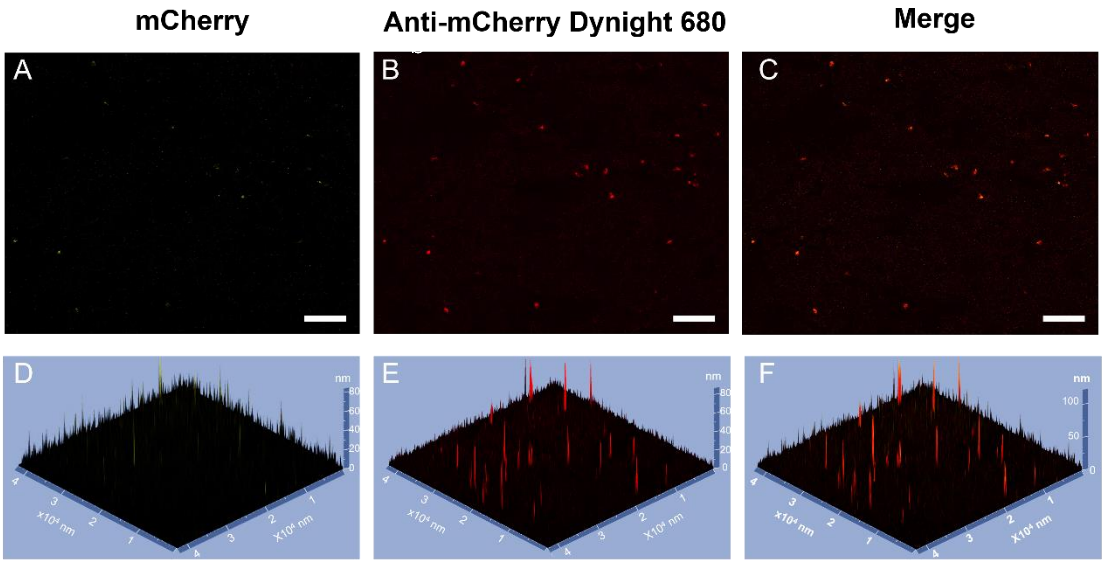

Figure S8. Colocalization of the mCherry and anti-mCherry antibody signals in mouse tumor tissue. A)-C) show representative histological images of 4T1 tumors with the expression of mCherry (A), the immunostaining of the mCherry protein (B), and the co-localization of both signals (C). D)-F) 2.5D topological representation of confocal laser microscopy images of mouse tumors after administration of mCherry mRNA and DOTAP/DOPE liposomes. D) The direct observation of mCherry protein fluorescence (yellow) only gains a low signal. E) Immunofluorescence staining with the additional mCherry antibody DyLight 680 (red) significantly improves the protein detection. F) The anti-mCherry antibody DyLight 680 signal co-localizes with the mCherry protein signal, turning it orange. Scale bar = 50 μm.

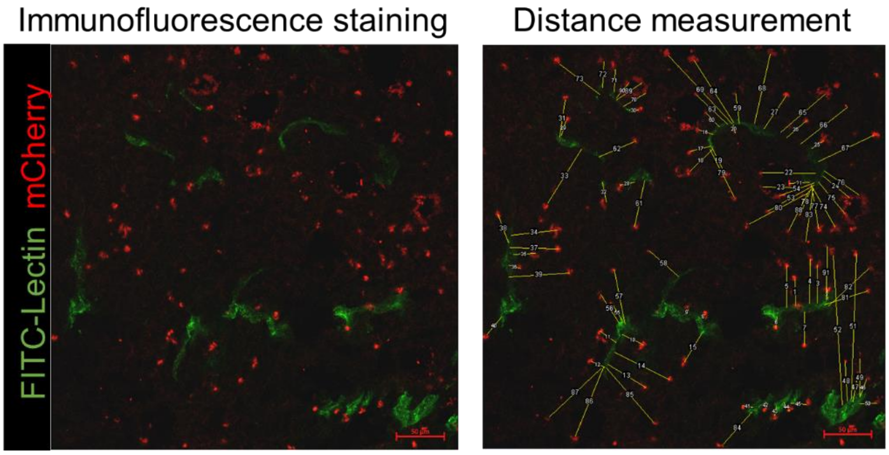

Figure S9. Representative example for measuring the distances between transfected cells and the closest vessel performed using ImageJ.

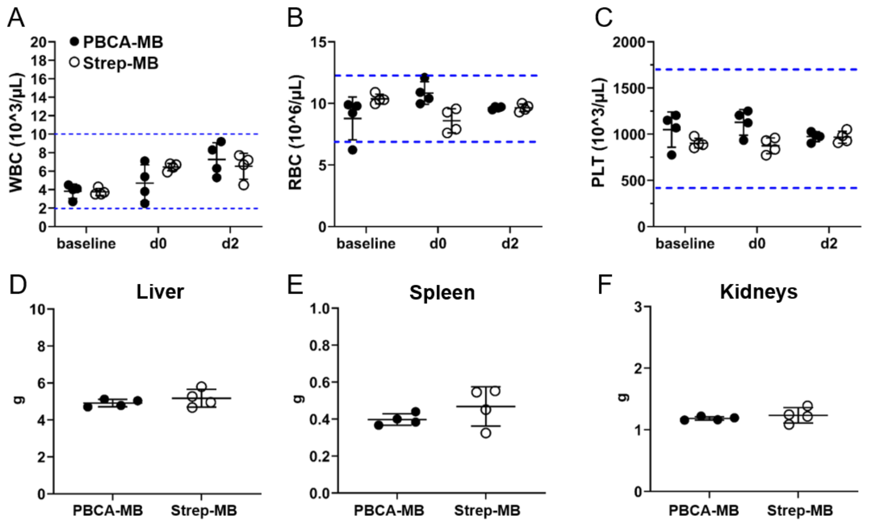

Figure S10. Blood values and organ weights of mice after administration of PBCA-MB and streptavidin coupled MB (Strep-MB). A-C) White blood cell counts (A), red blood cell counts (B), and platelet counts (C) before injection (baseline), directly after injection (d0), and 2 days after injection (d2) remained in the normal range (blue dotted lines; Normal values: white blood cells (WBC): 2~10 $\times 10^3$/µL; red blood cells (RBC): 6.93~12.24 $\times 10^6$/µL; platelets (PLT): 420~1698 $\times 10^3$/µL; Reference values origin from Charles River Laboratories; https://www.criver.com/sites/default/files/resources/doc_a/BALBcMouseClinicalPathologyData.pdf) and Practical Murine Hematopathology (PMID: 25926395)). D-F) Organ weights of mouse livers (D), spleens (E), and kidneys (F) show no alterations 2 days after MB injection.

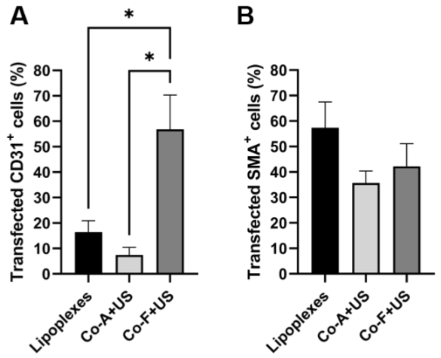

Figure S11. Quantification of the percentage of A) transfected endothelial cells and B) smooth muscle cells (SMA) based on the co-staining of CD31, SMA, and mCherry antibodies. The transfected endothelial cells (%) were calculated by dividing the number of CD31-positive and mCherry-positive cells by the total mCherry-positive cells. Co-formulation (Co-F) with US resulted in more transfected endothelial cells than lipoplexes alone or co-administration (Co-A). The transfected SMA cells (%) were also calculated by dividing the number of SMA-positive and mCherry-positive cells by the total mCherry-positive cells. *$p < 0.05$.
